# Ultrasensitive and Multiplexed Target Detection Strategy Based on Photocleavable Mass Tags and Mass Signal Amplification

**DOI:** 10.3390/nano15151170

**Published:** 2025-07-29

**Authors:** Seokhwan Ji, Jin-Gyu Na, Woon-Seok Yeo

**Affiliations:** Department of Bioscience and Biotechnology, Bio/Molecular Informatics Center, Konkuk University, Seoul 05029, Republic of Korea; ishjs00i@naver.com (S.J.); gooflovhoxjo@daum.net (J.-G.N.)

**Keywords:** hybridization chain reaction, mass spectrometry, mass tags, multiplexed detection, photocleavable, signal amplification

## Abstract

Co-infections pose significant challenges not only clinically, but also in terms of simultaneous diagnoses. The development of sensitive, multiplexed analytical platforms is critical for accurately detecting viral co-infections, particularly in complex biological environments. In this study, we present a mass spectrometry (MS)-based detection strategy employing a target-triggered hybridization chain reaction (HCR) to amplify signals and in situ photocleavable mass tags (PMTs) for the simultaneous detection of multiple targets. Hairpin DNAs modified with PMTs and immobilized loop structures on magnetic particles (Loop@MPs) were engineered for each target, and their hybridization and amplification efficiency was validated using native polyacrylamide gel electrophoresis (PAGE) and laser desorption/ionization MS (LDI-MS), with silica@gold core–shell hybrid (SiAu) nanoparticles being employed as an internal standard to ensure quantitative reliability. The system exhibited excellent sensitivity, with a detection limit of 415.12 amol for the hepatitis B virus (HBV) target and a dynamic range spanning from 1 fmol to 100 pmol. Quantitative analysis in fetal bovine serum confirmed high accuracy and precision, even under low-abundance conditions. Moreover, the system successfully and simultaneously detected multiple targets, i.e., HBV, human immunodeficiency virus (HIV), and hepatitis C virus (HCV), mixed in various ratios, demonstrating clear PMT signals for each. These findings establish our approach as a robust and reliable platform for ultrasensitive multiplexed detection, with strong potential for clinical and biomedical research.

## 1. Introduction

Recent advances in disease diagnostics have focused on achieving both high sensitivity and robust multiplexing to detect multiple targets at ultralow abundances [1,2]. Two amplification strategies have been generally adopted to enhance sensitivity: direct target amplification and transduced signal amplification. To detect oligonucleotide analytes, conventional enzyme-based direct target amplification techniques, such as the polymerase chain reaction (PCR) [3], rolling circle amplification (RCA) [4], and loop-mediated isothermal amplification (LAMP) [5], are widely used. Although these techniques offer excellent sensitivity and specificity with minimal sample requirements, they suffer from complex primer design, nonspecific amplification, enzyme instability, reliance on cold-chain logistics, and susceptibility to inhibitors in clinical specimens [6,7,8,9,10]. In parallel, transduced signal amplification techniques—including surface plasmon resonance (SPR), fluorescence, and electrochemiluminescence (ECL)-based amplification—improve detection without target replication, yet each faces its own limitations. SPR improves detection by capturing target molecules on gold nanostructures; however, nonspecific binding and surface contamination can compromise measurement accuracy [11]. Fluorescence amplification offers high sensitivity but is often limited by self-quenching effects and spectral overlap, leading to increased background signals and potential errors [12]. ECL utilizes redox reactions to generate strong signals [13,14]; however, it remains susceptible to interference from nonspecific reactions in complex sample matrices [15]. These techniques have primarily been applied in single-target systems for detecting individual targets. However, when extended to multiplexed detection, their performance often deteriorates because of intrinsic limitations [16]. For instance, SPR requires complex surface patterning and precise control for multiplexing [11], and fluorescence-based methods suffer from increased signal interference and self-quenching [12]. Similarly, ECL-based systems become vulnerable to overlapping electrochemical signals and nonspecific redox reactions in complex samples [17,18]. These limitations collectively affect the reliability, sensitivity, and scalability of conventional amplification techniques in multiplexed detection platforms.

Mass spectrometry (MS) has emerged as a powerful analytical tool in biomolecular analysis and various diagnostic applications [19,20,21]. Theoretically, MS can simultaneously detect an unlimited number of targets, providing unprecedented potential for comprehensive biomarker analysis. In particular, matrix-assisted laser desorption/ionization time-of-flight mass spectrometry (MALDI-TOF MS) is widely used as a rapid, simple-to-operate analytical tool owing to its high sensitivity and multiplexing capability [22]. However, MALDI approaches often suffer from poor quantitative reliability [23], the need for an organic matrix, and difficulty in detecting low-abundance macromolecules. To overcome these limitations, our group previously proposed a mass signal amplification strategy for quantitative and ultrasensitive macromolecule analysis using organic matrix-free LDI-TOF MS and gold nanoparticles (AuNPs) conjugated with numerous small reporter molecules, termed amplification tags (Am-tags) [24,25,26,27,28,29,30]. Despite its effectiveness, we found an inherent limitation of this approach for general use: the Am-tags must be conjugated to a solid support, specifically, gold surfaces. Therefore, we developed new mass tags that can generate ions via in situ photocleavage through laser irradiation in MS equipment. These in situ photocleavable mass tags (PMTs) are synthetically flexible, which allows a variety of masses to be obtained for multiplexing analysis, and contain a carboxylic acid group, which allows them to react with the various functional groups on nano-/micro-materials and (bio)molecules for common and general use [31]. Organic matrix-free LDI MS analysis demonstrated that PMTs generate in situ photocleaved cations upon laser irradiation, yielding high signal-to-noise (S/N) ratios. Quantitative evaluation confirmed the suitability of PMTs for multiplexed detection and target quantification. Furthermore, the PMTs exhibited sufficient stability, which supports their broad applicability in conjugation with various biomolecules and materials.

In this study, we employed PMTs to develop a novel MS-based detection system that integrates a target-triggered mass signal amplification method. Our work introduces an innovative enzyme-free and fluorescence-free signal amplification method, leveraging a hybridization chain reaction (HCR) [32,33] coupled with PMTs detected with mass spectrometry. Unlike traditional enzyme-dependent methods or fluorescence-based approaches, our method employs an HCR, thus avoiding issues associated with enzyme stability, complicated reaction conditions, or fluorescence signal quenching. Additionally, quantitative reliability, often a limitation of MS-based approaches, is effectively addressed by incorporating an internal standard (IS). By using hepatitis B virus (HBV) as a model system, we systematically validated the specificity, amplification efficiency, and quantitative reliability of our method in both buffer and complex matrices such as fetal bovine serum (FBS). Furthermore, the system was extended to include human immunodeficiency virus (HIV) and hepatitis C virus (HCV) targets, demonstrating successful multiplexed detection with clear mass-resolved signals corresponding to each target. This work highlights the potential of our strategy as a reliable and highly sensitive platform for multiplexed detection with broad applicability.

**Figure 1:**
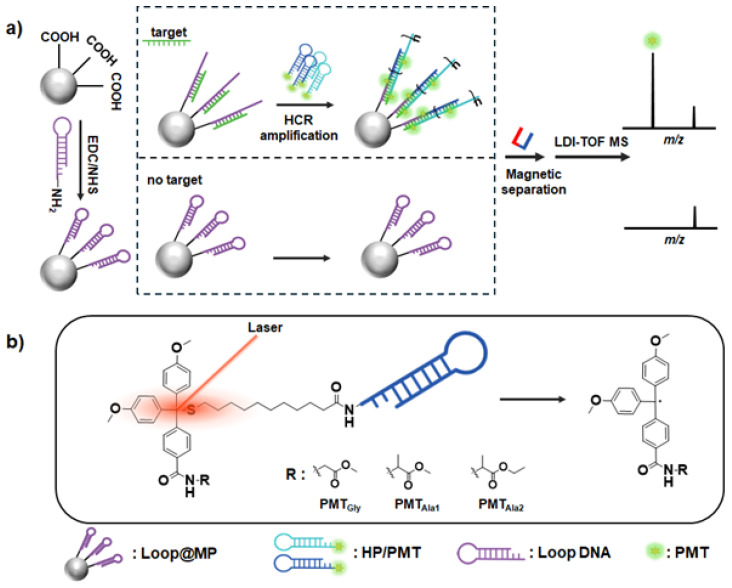
Schematic of our amplification strategy for oligonucleotide detection. (**a**) Target DNA triggers HCR amplification on MPs, which produces concatemer structures, which are analyzed with organic matrix-free LDI-TOF MS, resulting in enhanced mass signals for PMTs on HPs. (**b**) Structure of HP/PMT conjugates and in situ photocleavage reaction upon laser irradiation.

Figure 1a illustrates our experimental strategy for detecting specific pathogenic gene sequences. Carboxylic-acid-functionalized magnetic particles (MPs) are conjugated with amine-functionalized loop DNA with a carbodiimide coupling reaction, thus forming loop-DNA-conjugated MPs (Loop@MPs). In the presence of a target, the loop on the MPs is specifically unwound, and the HCR process ensues with two complementary hairpin DNAs (HPs) conjugated with PMTs (HP/PMTs), thus forming concatemers. Subsequent organic matrix-free LDI-TOF MS analysis then allows for the acquisition of PMT signals from the HP/PMTs in the concatemer, transducing the presence of the target into highly amplified MS signals. The structure of an HP/PMT and the laser-induced in situ photocleavage reaction are depicted in Figure 1b. In this study, we utilized three types of PMTs with different molecular weights, PMT_Gly_, PMT_Ala1_, and PMT_Ala2_; their structures are provided in the Supplementary Information (Figure S1).

## 2. Experimental Section

### 2.1. Materials

The carboxylic-acid-functionalized magnetic nanoparticles (MPs; particle size of 500 nm) were purchased from CD Bioparticles (Shirley, NY, USA), and all oligonucleotides used in this work were purchased from Integrated DNA Technologies, Inc. (Coralville, IA, USA). Acrylamide solution (40%) was purchased from Bio-Rad Laboratories, Inc. (Hercules, CA, USA), glycerol from Wako Pure Chemical Industries, Ltd. (Osaka, Japan), 10× TBE buffer from ForBioKorea Co., Ltd. (Seoul, Republic of Korea), *N*,*N*,*N’*,*N’*-Tetramethyl ethylenediamine (TEMED) from Biosesang (Yongin, Republic of Korea), and ammonium persulfate (APS) from Dyne Bio Inc. (Seongnam, Republic of Korea). The photocleavable mass tags (PMTs) were synthesized using a previously reported method [31]. An ethidium bromide (EtBr) solution, 1-ethyl-3-(3-dimethylaminopropyl) carbodiimide hydrochloride (EDC), *N*-hydroxy succinimide (NHS), *N*,*N*-dimethyl formamide (DMF), and phosphate buffer (PB) solution were purchased from Sigma-Aldrich (St. Louis, MO, USA), and fetal bovine serum (FBS) was purchased from Gibco. The silica@gold core–shell hybrid (SiAu) was prepared with a previously reported method [34].

### 2.2. Polyacrylamide Gel Electrophoresis (PAGE)

PAGE analysis was performed using a 12% polyacrylamide gel composed of 40% acrylamide solution, glycerol, TDW, 10× TBE buffer, APS, and TEMED. The loop DNA (12 μL, 500 nM), target DNA (12 μL, 10 μM), and HP DNA (12 μL, 1 μM) were incubated for the hybridization chain reaction (HCR) at room temperature for 2 h. The DNA samples were analyzed by electrophoresis in 1× TBE buffer at 120 V for 60 min, and EtBr was then employed to stain the gel.

### 2.3. Preparation of Loop-DNA-Conjugated MPs (Loop@MPs)

The amine-terminated loop DNA (20 μL, 1 μM in 10 mM PB at pH 7.4) was annealed at 95 °C for 5 min and then incubated at room temperature for 1 h to form a stable hairpin structure. The carboxylic-acid-functionalized MPs were washed three times with PB by using a magnetic separation device (MR-02, Thermo Fisher Scientific, Waltham, MA, USA), and the particle concentration was adjusted to 0.15 mg/mL. The MPs were reacted with EDC/NHS (100 μL each, 100 mM in PB) for 1 h and then washed once with PB. The resulting NHS-activated MPs were then resuspended in 180 μL of PB, and 20 μL of loop DNA was added. The mixture was incubated for 2 h and washed twice with PB, and the reaction was quenched with Tris buffer (20 μL, 1 M at pH 7.2) for 30 min. The resulting loop-DNA-conjugated MPs (Loop@MPs) were redispersed in PB and stored at 4 °C for further use.

### 2.4. Preparation of Hairpin DNA-PMT Conjugates (HP/PMTs)

The PMT solution (40 μL, 10 mM in DMF) was reacted with EDC/NHS (10 μL each, 100 mM in DMF) for 1 h at room temperature, followed by the addition of HP DNA (20 μL, 100 μM in PB) and 40 μL of PB. After incubation overnight, the mixture was washed twice with dichloromethane to remove organic matter, yielding the HP/PMTs.

### 2.5. Detection of Oligonucleotide Targets

To detect the target DNA, the Loop@MPs were suspended in 180 μL of PBS (400 mM NaCl at pH 7.4) and treated with the target DNA (20 μL, 1 μM in PB) for 2 h, followed by the HP/PMTs. Then, the mixture was washed twice with PB and additionally with deionized water. The HCR products on the MPs were thermally released at 95 °C for 5 min, and the supernatant (5 μL) was collected and analyzed by using LDI-MS with SiAu (5 μL, 10 μg/mL in ethanol) as the matrix.

### 2.6. Detection of Multiple Oligonucleotide Targets

To simultaneously detect multiple target DNAs, three distinct types of Loop@MPs (Loop1@MPs for hepatitis B virus (HBV), Loop2@MPs for human immunodeficiency virus (HIV), and Loop3@MPs for hepatitis C virus (HCV)) were mixed in 180 μL of PBS. The HIV, HBV, and HCV target mixtures in different ratios, 1:1:1, 1:0:1, 1:1:0, and 0:1:1 (20 μL, 1 μM each in PB), were incubated with the mixture of Loop@MPs for 2 h. Subsequently, the mixture of six types of HP/PMTs (HP1,2/PMT_Ala1_ for HBV; HP3,4/PMT_Gly_ for HIV; and HP5,6/PMT_Ala2_ for HCV) was introduced to initiate the 2 h HCR reaction, after which the mixture was washed twice with PB and additionally with deionized water. The HCR products on the MPs were thermally released at 95 °C for 5 min, and the supernatant (5 μL) was collected and analyzed by using laser desorption/ionization mass spectrometry (LDI-MS) with SiAu (5 μL, 10 μg/mL in ethanol) as the matrix.

### 2.7. LDI-MS Analysis

MS analysis was performed with an Autoflex III matrix-assisted laser desorption/ionization time-of-flight (MALDI-TOF) mass spectrometer (Bruker Daltonics, Bremen, Germany) equipped with a smart-beam laser as an ionization source. The spectra were obtained in positive mode at an accelerating voltage of 19 kV and a repetition rate of 75 Hz, and each comprised an average of 500 shots.

### 2.8. Oligonucleotide Detection in Spiked Samples

To detect the HBV target in the complex samples, a total of four target DNA solutions (1 or 10 fmol, 20 μL) were prepared in 1% or 5% FBS. Loop1@MPs were suspended in 180 μL of PBS, and each spiked FBS sample was added to the suspension. The mixture was incubated for 2 h, followed by the addition of HP1/PMT_Ala1_ and HP2/PMT_Ala1_. Then, the mixture was washed twice with PB and additionally with deionized water. The HCR products on the MPs were thermally released at 95 °C for 5 min, and the supernatant (5 μL) was collected and analyzed by using LDI-MS with SiAu (5 μL, 10 μg/mL in ethanol) as the matrix.

## 3. Results and Discussion

### 3.1. Validation of Target-Triggered Signal Amplification

**Figure 2:**
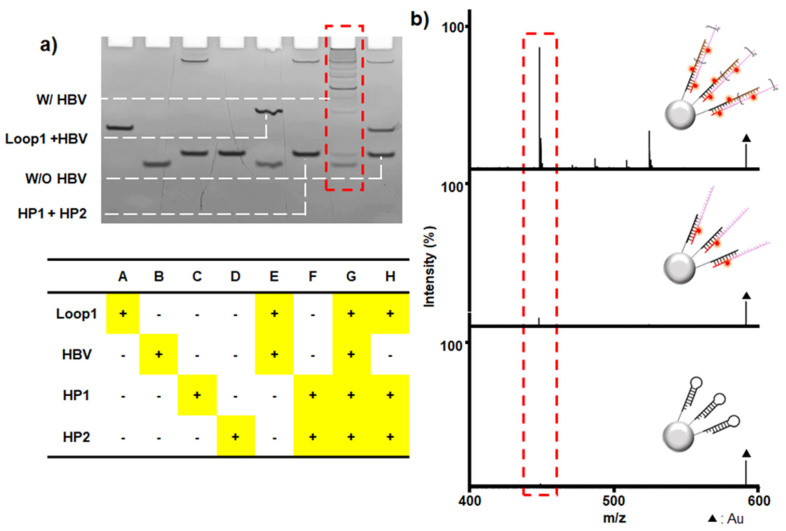
Verification of the HCR specificity of the designed oligonucleotides for cDNA HBV as a target. (**a**) Native PAGE analysis verifying target-specific HCR initiation and concatemer formation. (**b**) LDI-MS analysis using each HP/PMTAla1 and Loop@MPs under different conditions: complete amplification in the presence of a target and both HP1 and HP2 (**top**), incomplete amplification in the absence of HP2/PMTAla1 (**middle**), and no initiation of the HCR without a target (**bottom**). SiAu was used as a matrix.

To validate our strategy, we examined a specific nucleotide sequence of HBV, a representative pathogenic DNA virus requiring rapid and accurate detection, as a model system. For this approach, a complementary DNA (cDNA)-based detection system consisting of loop 1, HP1, and HP2 DNA was designed; detailed sequence information is provided in Table S1. First, native polyacrylamide gel electrophoresis (PAGE) analysis was performed to evaluate the binding specificity and amplification efficiency of the designed DNA sequences (Figure 2a). For the mixture of loop 1, the target, and both HP1 and HP2, lane G exhibited ladder-like bands with high molecular weights, indicating that the HCR proceeded to form concatemers. In lane E (the mixture of loop 1 and the target, without HP1 and HP2), the complementary binding between loop 1 and the target was confirmed, and lane F (HP1 and HP2) confirmed no self-assembly between HP1 and HP2. However, in the absence of the target, HP1 and HP2 did not bind to loop 1, implying that the loop structure was conserved, as shown in lane H. These results confirm that the target opens loop 1, triggering alternating HP binding and concatemer formation, thus validating our target-specific HCR design.

We next employed the designed DNA to further verify our mass signal amplification strategy described in Figure 1a. To fabricate Loop1@MPs, carboxylated MPs were covalently linked to loop 1 with carbodiimide coupling reactions, where the optimal loading amount of loop 1 was determined based on the manufacturer’s protocol. In this experiment, PMT_Ala1_ was used to prepare HP/PMT conjugates. HPs modified with amine groups were conjugated to the carboxylic acid moiety of the PMTs via carbodiimide coupling reactions. After incubating the HBV target (1 pmol) with the Loop1@MPs, HP1/PMT_Ala1_, and HP2/PMT_Ala1_, the unreacted target and both HP1/ and HP2/PMT_Ala1_ were removed with thorough washing. The DNA complexes were thermally released from the MPs, followed by magnetic separation, and the supernatant was analyzed using organic matrix-free LDI-MS, which revealed a clear, high-intensity peak corresponding to PMT_Ala1_ (Figure 2b (top)). In a control experiment where only HP1/PMT_Ala1_ was used, without HP2/PMT_Ala1_, the major peak was significantly less intense (Figure 2b (middle)). Moreover, this peak was negligible for HP1/PMT_Ala1_ and HP2/PMT_Ala1_ without the target (Figure 2b (bottom)). These results clearly demonstrate that target-induced loop opening triggers the complementary binding of HP1/PMT_Ala1_ and HP2/PMT_Ala1_ to form concatemers, thereby amplifying the MS signal. Note that our HCR-based amplification strategy depends on target-induced loop opening; therefore, mutations or sequence variations may affect loop-opening efficiency and detection specificity. We plan to evaluate these effects in future investigations. A direct MS analysis of the MPs containing concatemers was initially performed to observe the amplified PMT signals, which showed high background signals stemming from the MPs, thus not only suppressing the PMT signals but also causing them to overlap (Figure S2). Previously, we found that silica@gold core–shell hybrid NPs (SiAu NPs) greatly enhanced the efficiency of the desorption/ionization process by laser irradiation, producing very low background signals [34]. Hence, we utilized SiAu for this study.

### 3.2. Assessment of SiAu NPs as an Internal Standard (IS)

**Figure 3:**
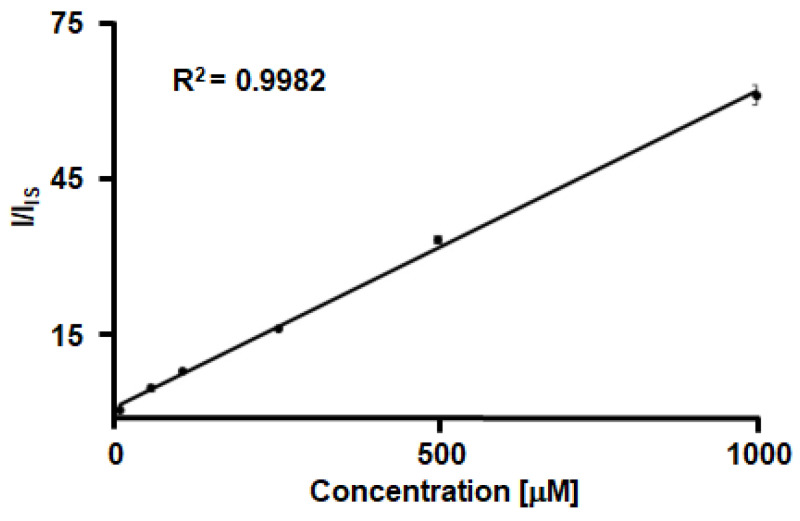
Evaluation of SiAu as the IS for quantitative analysis with LDI-MS. The error bars represent the standard deviation (n = 3).

Conventional LDI-MS-based analysis often exhibits poor quantitative reliability because of the weak correlation between the intensity and the actual amount of an analyte. To address this limitation, the same amount of a chemical species, known as an IS, is added to samples to correct the sample-to-sample variations in MS intensity during analysis. In our study, SiAu was utilized as the IS after evaluating its applicability for quantitative analysis. Specifically, PMT_Ala1_ was prepared in various concentrations, 1, 50, 100, 250, 500, and 1000 μM, and each solution (5 μL) was dropped onto the LDI plate and dried, followed by the addition of SiAu (5 μL) at a concentration of 10 μg/mL. The MS analysis showed that the peak intensity ratio (I/I_IS_) increased linearly with the PMT_Ala1_ concentration, confirming the reliability of SiAu as the IS for quantitative analysis (Figure 3).

### 3.3. Optimization of Experimental Conditions

**Figure 4:**
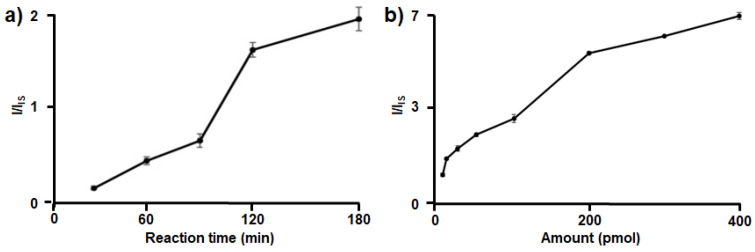
Determination of the optimized HCR reaction time and amount of HPs based on MS analysis using an IS. (**a**) The mass signal increased over time and reached a plateau after 120 min. (**b**) The mass signal increased as the amount of HPs increased, leveling off above 200 pmol. The error bars represent the standard deviation (n = 3).

Next, the experimental conditions were optimized using SiAu as the IS. To monitor the intensity changes during the HCR, the amounts of loop 1 and the target were fixed at 1 pmol, and 50 pmol each of HP1/PMT_Ala1_ and HP2/PMT_Ala1_ was used. As the HCR reaction time increased, the I/I_IS_ ratio increased significantly up to 120 min; thereafter, the rate of increase dropped to approximately 16%, indicating that extending the reaction time over 120 min had a limited effect on signal amplification (Figure 4a). To optimize the amount of HPs, the effect of varying HP1/PMT_Ala1_ and HP2/PMT_Ala1_ within the range of 5–400 pmol was examined with 1 pmol of both loop 1 and the target for a reaction time of 120 min. The results show that the I/I_IS_ ratio increased with higher amounts of HP1/PMT_Ala1_ and HP2/PMT_Ala1_; however, further enhancement was negligible above 200 pmol (Figure 4b). Considering the amplification effectiveness, cost, and assay time of our method, we thereafter used a reaction time of 120 min with 200 pmol of each HP/PMT_Ala1_.

### 3.4. Evaluation of Quantitative Aspects

**Figure 5:**
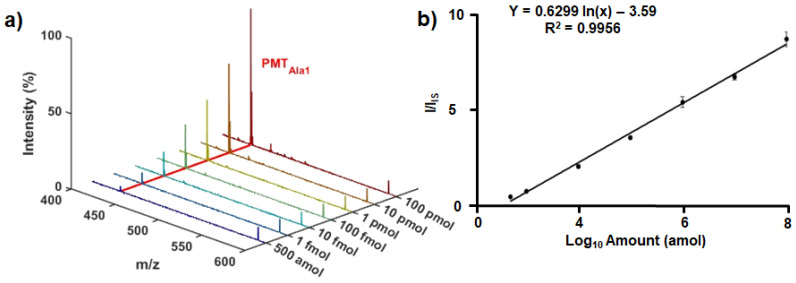
Assessment of the quantitative reliability of our strategy. (**a**) Representative LDI-MS spectra showing a progressive increase in the PMT_Ala1_ signal with the increase in the amount of target. (**b**) Calibration curve of I/I_IS_ plotted against the logarithm of the target amount. The error bars represent the standard deviation (n = 3).

Next, the reliability of the quantitative aspect was assessed by constructing a calibration curve using the optimized conditions described above. The target amount was adjusted from 500 amol to 100 pmol, and LDI-MS analysis was performed using SiAu as the IS. In the representative spectra normalized using the IS peak shown in Figure 5a, the PMT_Ala1_ peak clearly increased with the increase in the amount of the target. The calibration curve derived from the MS spectra displayed outstanding linearity (R^2^ = 0.9956) between the I/I_IS_ ratio and the logarithm of the target amount (Figure 5b), thereby validating the system’s quantitative reliability. The observed standard deviations were minimal—±0.025 at 500 amol and ±0.601 at 100 pmol—implying the method’s excellent reproducibility, as well as its wide dynamic range, from 1 fmol to 100 pmol. Based on this correlation, the limit of detection (LOD) for the HBV target was calculated to be 415.12 amol, which is comparable to or even better than those of previous reports (Table S2), implying that the system offers outstanding sensitivity for quantifying ultralow-abundance target DNA. While the calibration LOD of our MS-HCR platform was determined in buffer, its effective sensitivity in real samples will be reduced by serum dilution. Moreover, recoverable viral material may further decrease during sample preparation if particles remain cell-associated or membrane-bound. Further work will systematically investigate these effects, optimize sample preparation protocols (e.g., direct plasma analysis and cell lysis), and determine the true LOD in patient-derived specimens.

### 3.5. Target Quantification in Spiked Serum Samples

To evaluate the practical applicability of the developed detection system in real sample environments, we also quantified the target in spiked samples. Four target DNA solutions were prepared at 1 or 10 fmol in 1% or 5% FBS, and the same experimental protocol described above was applied. By comparing the intensity of the peaks for PMT_Ala1_ and the IS, the target could be quantified using the constructed calibration curve, demonstrating both high accuracy and precision under various conditions, as shown in Table 1. Note that the precision and accuracy were slightly reduced for the lower-abundance target in the higher-concentration FBS samples (i.e., 1 fmol and 5% FBS). These findings confirm that our method facilitates the accurate and sensitive quantification of the targets present in complex samples in low abundance.

### 3.6. Evaluation of Multiplexed Detection Capability and Specificity

**Figure 6:**
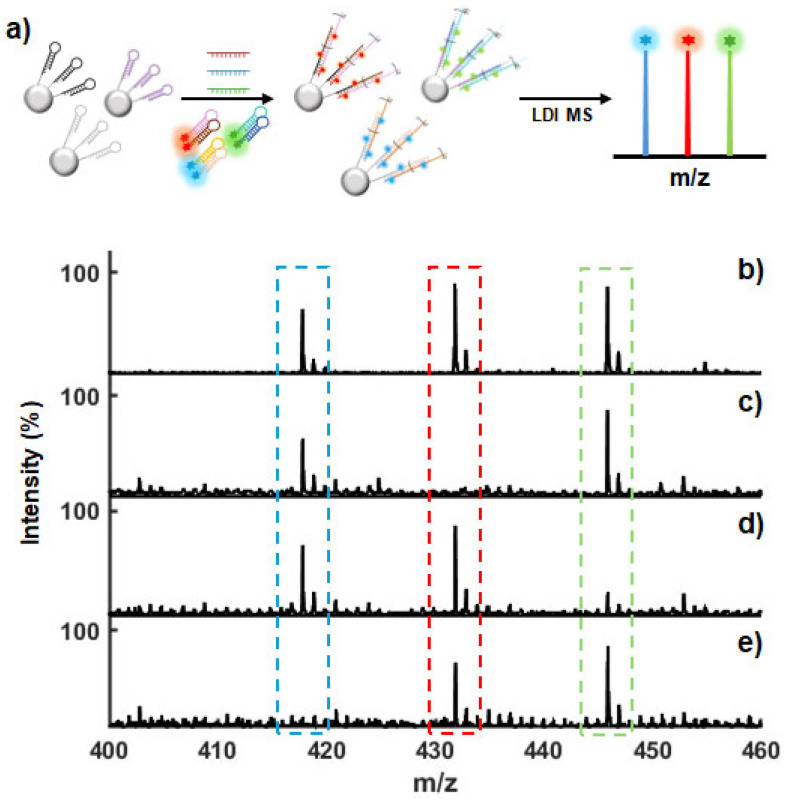
Validation of the multiplexing capability of our strategy. (**a**) Schematic overview of the multiplexing strategy for simultaneously detecting three different targets. (**b**–**e**) Mass spectra for detecting HIV, HBV, and HCV target mixtures in different ratios, i.e., (**b**) 1:1:1; (**c**) 1:0:1; (**d**) 1:1:0; and (**e**) 0:1:1, using three distinct PMTs.

Finally, to further verify the applicability of our system, we assessed its reliability in the simultaneous detection of multiple targets, which is essential to accurately and efficiently diagnosing infections. For instance, patients with HIV and HCV are often co-infected with HBV, and such co-infections pose significant clinical challenges [35,36,37,38]. Therefore, the development of a system capable of simultaneously detecting these viruses is critical to accurately diagnosing blood-borne infections. Hence, HIV and HCV targets were also included in the analysis to further evaluate the performance of our system. For this approach, cDNA-based detection systems for HIV and HCV were designed, each consisting of loop and HP DNA; detailed sequence information is provided in Table S1. PAGE analyses were performed to examine the binding specificity and amplification efficiency of the designed DNA sequences, verifying the target-initiated specifically formed concatemer structures (Figure S3). To validate MS detection of other targets, we prepared the HP3/PMT_Gly_ and HP4/PMT_Gly_ conjugates and Loop2@MPs for HIV and the HP5/PMT_Ala2_ and HP6/PMT_Ala2_ conjugates and Loop3@MPs for HCV. After incubating each of the targets with the Loop@MPs and HP/PMTs, the DNA complexes were thermally released from the MPs and analyzed with LDI-MS. As shown in Figure S4, high-intensity peaks corresponding to PMT_Gly_ for the HIV target and PMT_Ala2_ for the HCV target clearly appeared, whereas control experiments using only one type of HP/PMT or conducted without the target resulted in only trace peaks for the PMTs. To simultaneously detect the three targets, three distinct Loop@MPs, each corresponding to a specific target (Loop1@MPs for HBV, Loop2@MPs for HIV, and Loop3@MPs for HCV) were mixed in equal amounts, followed by the incubation of six distinct HP/PMTs, each corresponding to a specific target (HP1/PMT_Ala1_ and HP2/PMT_Ala1_ for HBV, HP3/PMT_Gly_ and HP4/PMT_Gly_ for HIV, and HP5/PMT_Ala2_ and HP6/PMT_Ala2_ for HCV) (Figure 6a). The MS spectra for the analysis of the HIV, HBV, and HCV target mixtures in different ratios, 1:1:1, 1:0:1, 1:1:0, and 0:1:1, showed either three or two distinct major peaks corresponding to the PMT molecules conjugated to the HP DNA targeting a specific analyte (Figure 6b–e). These results confirm our method’s efficacy for both single- and multi-target detection, validating its reliability as a multiplexed platform.

**Figure 7:**
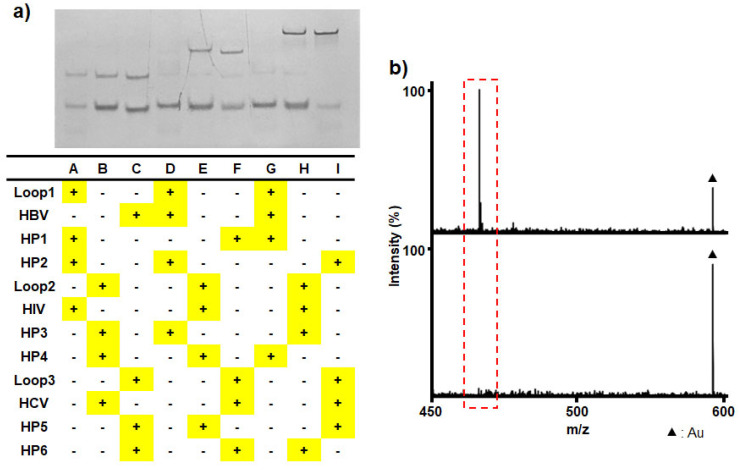
Validation of nonspecific interactions and probe–probe interference. (**a**) Native PAGE analysis under various mismatch conditions. Loops incubated with non-cognate targets remained closed due to sequence mismatch (lanes A–C). Loops activated by cognate targets in the presence of mismatched hairpins (HP2, -4, and -6) did not yield concatemer formation (lanes D–F). Loops activated by cognate targets with the correct initial hairpins (HP1, -3, and -5) but lacking complementary hairpins (HP2, -4, and -6) also failed to produce concatemers (lanes G–I). (**b**) LDI-MS analysis of the HBV detection module in the presence of nonspecific targets. A distinct PMT_Ala1_ peak was clearly observed when all three targets (HBV, HCV, and HIV) were present, whereas no PMT_Ala1_ peak was detected in the absence of the HBV target (with only HCV and HIV present) (**bottom**), confirming negligible cross-reactivity.

Furthermore, potential nonspecific interactions in the presence of nonspecific oligonucleotides and probe–probe interference were evaluated using native PAGE analysis and mass spectrometry. As shown in Figure 7a, native PAGE revealed no concatemer bands, indicating minimal cross-reactivity and confirming the high specificity of our system. The loops incubated with non-cognate targets remained closed due to sequence mismatch (lanes A–C), and those activated by cognate targets in the presence of mismatched hairpins (HP2, -4, and -6) did not produce concatemers (lanes D–F). Similarly, the loops activated by cognate targets with the correct initial hairpins (HP1, -3, and -5) failed to form concatemers in the absence of the complementary hairpins (HP2, -4, and -6) (lanes G–I). MS analysis further validated the absence of cross-reactivity and confirmed the specificity of the HBV detection module (Loop1@MPs and HP1,2/PMT_Ala1_). A distinct PMT_Ala1_ peak was clearly observed when all three targets (HBV, HCV, and HIV) were present, indicating selective detection of the HBV target without interference from non-cognate sequences (Figure 7b (top)). In contrast, no PMT_Ala1_ peak was detected in the absence of the HBV target (with only HCV and HIV present), confirming minimal cross-reactivity (Figure 7b (bottom)). Similar results were also obtained with the HCV and HIV detection modules, demonstrating consistent specificity across different targets (Figure S5). Taken together, these findings underscore the platform’s strong specificity, demonstrating negligible cross-reactivity in the context of complex multi-analyte environments.

## 4. Conclusions

We successfully developed a novel HCR-based mass signal amplification system optimized for detecting DNA targets by employing in situ PMTs and LDI-MS. With SiAu as the IS, the system achieved excellent linearity across a broad dynamic range, demonstrating high sensitivity, selectivity, and quantitative reliability. The multiplexed detection of three targets for viral pathogens was achieved with clearly amplified MS signals, confirming the versatility of the platform. Although validation with real clinical samples—which would require additional optimization and tailored probe design—could further enhance clinical applicability, we believe that our results demonstrate the potential of the system as an efficient and robust diagnostic tool, paving the way for future clinical and biomedical applications.

## Figures and Tables

**Table 1:** Measurement of target DNA levels in spiked FBS samples. Values are based on three independent experiments.

Condition	Sample Amount	Expected (I/I_IS_)	Observed (I/I_IS_)	Precision (CV, %)	Accuracy (%)
1% FBS	1 fmol	0.761	0.762	7.5	100.2
1% FBS	10 fmol	2.212	2.196	8.5	99.3
5% FBS	1 fmol	0.761	0.697	13.4	91.6
5% FBS	10 fmol	2.212	2.149	5.4	97.2

## Data Availability

The data presented in this study are available upon request from the corresponding author.

## Supplementary Materials

The following supporting information can be downloaded at https://www.mdpi.com/article/10.3390/nano15151170/s1: Figure S1: Structures and molecular weights of the three detected ions for the PMTs; Table S1: Oligonucleotide sequences used in this study; Figure S2: LDI-MS analysis of HCR products on MPs; Table S2: LODs of representative methods for detecting oligonucleotides; Figure S3: Native PAGE analysis confirming target-specific initiation using oligonucleotides designed for HIV and HCV; Figure S4: LDI-MS analysis confirming target-specific HCR amplification for HIV and HCV. Figure S5. LDI-MS analysis of the target detection module in the presence of non-specific targets. Refs. [12,39,40,41,42] are cited in the supplementary materials.
