# Supplementary material for: Ultrasensitive and Multiplexed Target Detection Strategy Based on Photocleavable Mass Tags and Mass Signal Amplification
Source: Nanomaterials (Basel). 2025 Jul 29;15(15):1170. doi: 10.3390/nano15151170 (PMC12348162; doi:10.3390/nano15151170)
Supplement: Supplementary file 1 [file nanomaterials-15-01170-s001.zip › nanomaterials-3659876-supplementary.pdf]

Ultrasensitive and Multiplexed Target Detection Strategy Based on Photocleavable Mass Tags and Mass Signal Amplification

Seokhwan Ji, Jin-Gyu Na and Woon-Seok Yeo *

Department of Bioscience and Biotechnology, Bio/Molecular Informatics Center, Konkuk University, Seoul 05029,
Republic of Korea

* Corresponding authors.

E-mail addresses: wsyeo@konkuk.ac.kr (W.-S. Yeo)

Ion	Structure	M.W
Gly	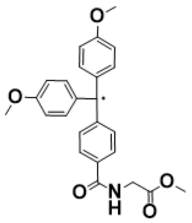	418.46
Ala1	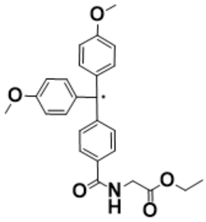	432.49
Ala2	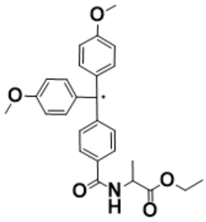	446.51

Figure S1. Structures and molecular weights of the three detected ions for the PMTs used in this study.

Table S1. Oligonucleotide sequences used in this study.

Name	Sequence (5′-3′)
Loop1	NH_2/TTT TTT TTT TTG GCT TTC AGT TAT ATG GAT GAT GTG GTA TTC CCC TCA TAC CAC ATC ATC CAT
HBV	ATA CCA CAT CAT CCA TAT AAC TGA AAG CCA
HP1	NH_2/ATA CCA CAT CAT CCA TAA ACC AAA TGG ATG ATG TGG TAT GAG GGG A
HP2	NH_2/TTG GTT TAT GGA TGA TGT GGT ATT CCC CTC ATA CCA CAT CAT CCA T
Loop2	NH_2/TTT TTT TTT TTT AAA GGA CCA GGC GCA ACT AAA TTC ATG GTC CCC TCT TCC CAT GAA TTT AGT TG
HIV	CCA TGA ATT TAG TTG CGC CTG GTC CTT TAA
HP3	NH_2/CCA TGA ATT TAG TTG AAA CCA ACA ACT AAA TTC ATG GGA AGA GGG GA
HP4	NH_2/TTG GTT TCA ACT AAA TTC ATG GTC CCC TCT TCC CAT GAA TTT AGT TG
Loop3	NH_2/TTT TTT TTT TTC GTG GAT AAA CCC GCT CAA TGC CTG GAG ATT CCC CTC TTC ATC TCC AGG CAT TGA G
HCV	ATC TCC AGG CAT TGA GCG GGT TTA TCC ACG A
HP5	NH_2/CTT CAT CTC CAG GCA TTG AGA AAC CAA CTC AAT GCC TGG AGA TGA AGA GGG GA
HP6	NH_2/TTG GTT TCT CAA TGC CTG GAG ATG AAG TCC CCT CTT CAT CTC CAG GCA TTG AG

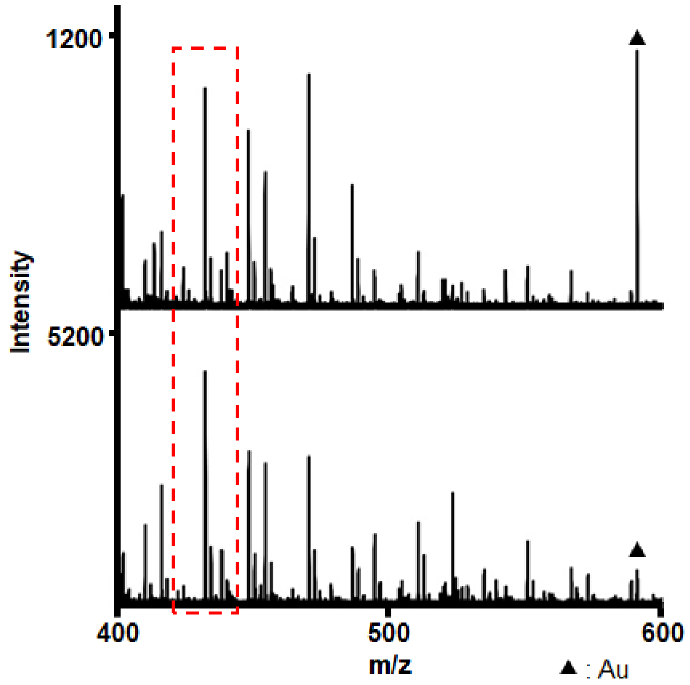

Figure S2. LDI-MS analysis of HCR products on MPs; MPs without loop DNA (top) and MPs after HCR amplification (bottom).

Table S2. Limits of detection (LODs) of representative methods for detecting oligonucleotides using various detection strategies.

**Method**	**Target**	**LOD**	**References**
ECL	Survivin mRNA	3 fmol	*Talanta* **2017**, *175,* 121-126
Fluorescence	HIV	55.8 fmol	*Anal. Chem* **2013**, *85*(24), 11929-11935
	HBV	50.4 fmol	
	HCV	52.2 fmol	
	miRNA-21	1 fmol	*Anal. Chem* **2023**, *95*(49), 18199–18206
	PCB72/106	1.8 fmol	*Anal. Chem* **2018**, *90*(16), 9936–9942
MS	miRNA-21	930 amol	*Nano Lett* **2023**, *23*(5), 1820–1829
	HBV	415.1 amol	This study

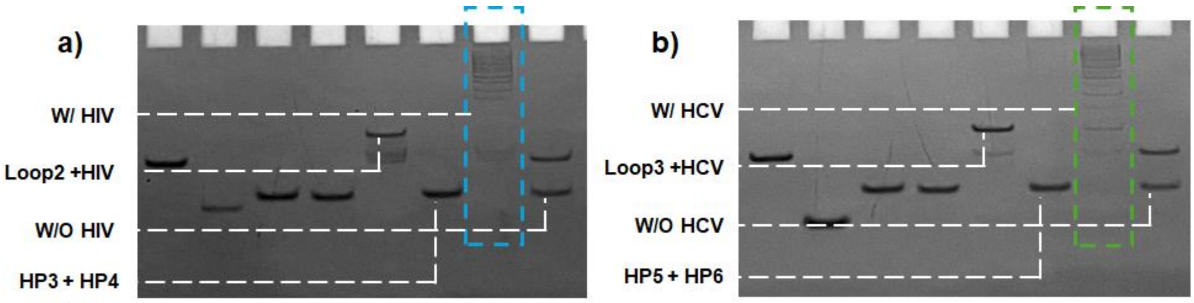

	A	B	C	D	E	F	G	H
Loop2	+	-	-	-	+	-	+	+
HIV	-	+	-	-	+	-	+	-
HP3	-	-	+	-	-	+	+	+
HP4	-	-	-	+	-	+	+	+

	A	B	C	D	E	F	G	H
Loop3	+	-	-	-	+	-	+	+
HCV	-	+	-	-	+	-	+	-
HP5	-	-	+	-	-	+	+	+
HP6	-	-	-	+	-	+	+	+

Figure S3. Native PAGE analysis confirming target-specific initiation using oligonucleotides designed for a) HIV and b) HCV.

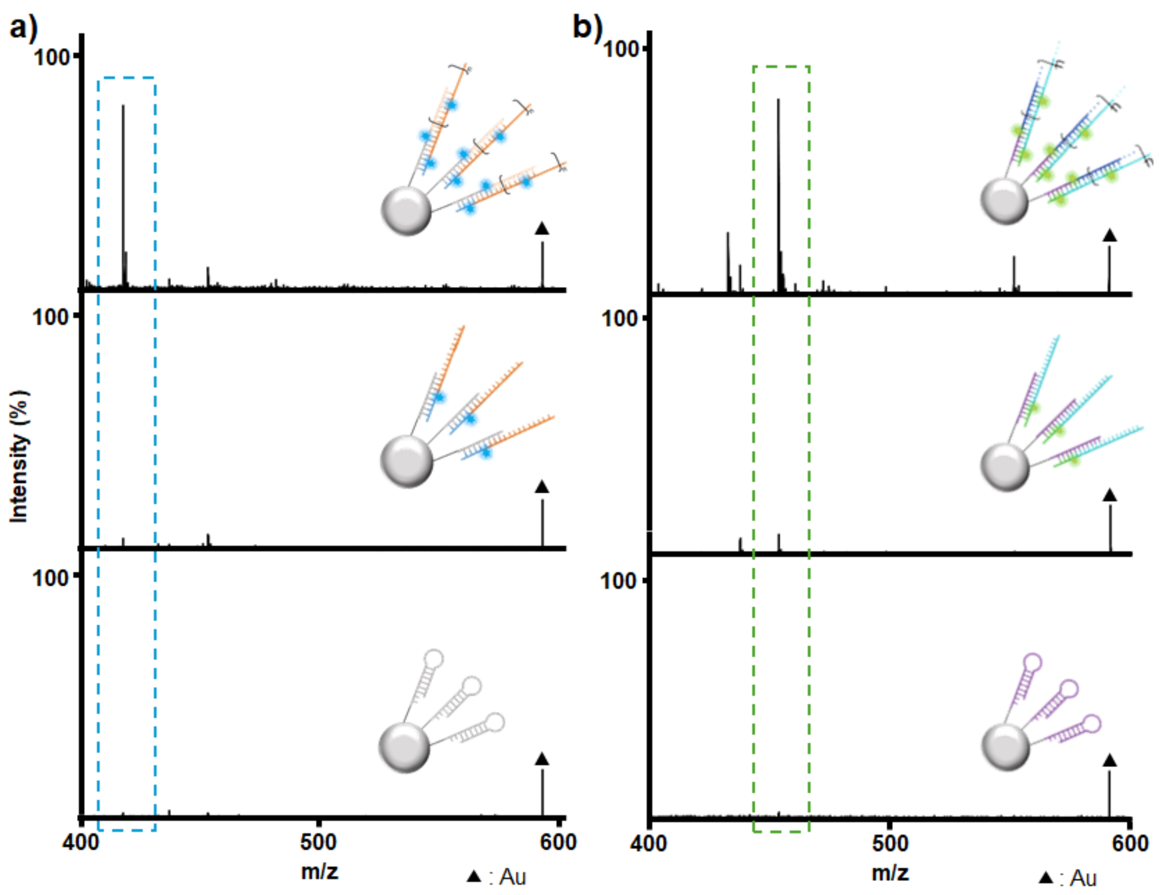

Figure S4. LDI-MS analysis confirming target-specific HCR amplification using a) Loop2@MPs with HP3/PMT$_{Gly}$ and HP4/PMT$_{Gly}$ for HIV and b) Loop3@MPs with HP5/PMT$_{Ala2}$ and HP6/PMT$_{Ala2}$ for HCV under different conditions; complete reaction in the presence of the target and both HPs (top), incomplete amplification in the absence of the HP3/PMT$_{Gly}$ or HP5/PMT$_{Ala2}$ (middle), and no HCR initiation without the target (bottom).

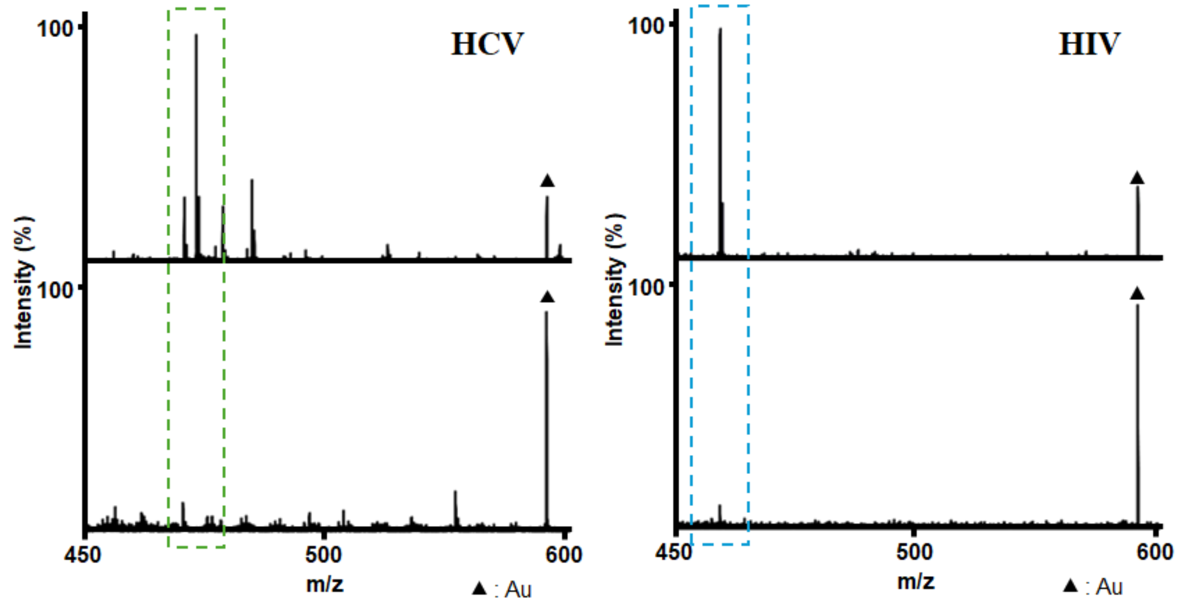

Figure S5. LDI-MS analysis of the target detection module in the presence of non-specific targets. A distinct PMT peak was observed when all three targets (HBV, HCV, and HIV) were present (top), indicating specific detection of HCV and HIV targets. In contrast, no PMT peak was detected in the absence of the target (bottom), confirming negligible cross-reactivity.
